# Editorial: Biosynthetic resorbable meshes: a New frontier in abdominal wall hernia repair

**DOI:** 10.3389/fsurg.2026.1961336

**Published:** 2026-08-20

**Authors:** Jose Bueno-Lledo, Frederik Berrevoet, John Fischer, J. Scott Roth

**Affiliations:** 1 Hospital Universitari I Politecnic La Fe, Valencia, Spain; 2 Department for General and HPB Surgery and Liver Transplantation, Ghent University Hospital, Ghent, Belgium; 3 Division of Plastic Surgery, Department of Surgery, University of Pennsylvania Health System, Philadelphia, PA, United States; 4 Division of General, Endocrine and Metabolic Surgery, Department of Surgery, University of Kentucky, Lexington, KY, United States

**Keywords:** abdominal wall surgery, absorbable mesh, biosynthetic mesh, hernia, hernia repair, mesh, synthetic mesh

Long-term absorbable biosynthetic meshes (LTABMs) have become a relevant addition to the armamentarium of abdominal wall surgeons, due to their practical position as a compromise between biologic scaffolds and permanent synthetics. Their appeal lies in the possibility of providing temporary mechanical support during the critical phases of wound healing while avoiding a lifelong implanted foreign body. For abdominal wall surgeons facing increasingly complex patients, this concept is intuitively attractive and clinically consequential.

Obesity, diabetes, smoking, prior mesh infection, stomas, multiple laparotomies, immunosuppression, and contamination have transformed hernia surgery into a field where the biological environment is as important as the fascial defect itself (1). In these settings, the traditional binary choice between permanent synthetic and biologic mesh has often proven unsatisfactory. Permanent synthetics offer strength and durability, but concerns persist regarding chronic infection, fistulization, pain, and the consequences of an implanted material that cannot be removed easily once incorporated. Biologic meshes, in contrast, were introduced as a solution for contaminated or high-risk fields, yet long-term durability has been inconsistent and their cost remains a major obstacle to routine use (2).

Several properties explain the growing interest in LTABMs. First, they offer a prolonged strength-retention profile intended to protect the repair through the biologically vulnerable phases of healing. The P4HB literature describes a scaffold with flexibility, handling characteristics, and durability that make it suitable for ventral and incisional hernia repair, while allowing eventual degradation into naturally occurring metabolites (3). Second, these meshes appear to support constructive remodeling rather than merely passive scar encapsulation. Preclinical data summarized in recent reviews show collagen deposition, vascular ingrowth, and progressive replacement of scaffold material by host tissue, with the hope that the final repair will depend more on biologically integrated tissue than on a permanent prosthesis. Third, the inflammatory profile may differ from that of conventional permanent synthetics. Experimental work has suggested a host response compatible with tissue remodeling and less persistent inflammatory activity than some traditional materials, although direct clinical translation remains difficult because comparative human histologic data are limited (4).

The strongest clinical argument for these meshes is their use in the gray zone of abdominal wall reconstruction. In a straightforward clean retromuscular repair of a low-risk patient, permanent synthetic mesh remains an efficient and durable option with a strong track record. The value of these prostheses becomes more evident when the field is less ideal, the host is less forgiving, or the consequences of a permanent implant are potentially greater. This includes patients with prior wound events, a history of mesh infection, concomitant stoma, immunosuppression, obesity, diabetes, smoking, or a clean-contaminated or contaminated operative field (5).

Available clinical studies support this selective enthusiasm more than any universal recommendation. A recent scoping review identified 79 peer-reviewed studies, including 50 clinical studies, and reported promising outcomes across hernia repair and soft tissue reconstruction, while also emphasizing the need for randomized trials and better definition of indications (Deeken et al.). These data suggest that the field has progressed beyond anecdotal experience, but not yet to definitive evidence.

Long-term follow-up is particularly important because a mesh that disappears must ultimately be judged after it is gone. In clean wound ventral hernia repair, long-term outcomes with LTABMs have been reported as acceptable, with recurrence rates up to 20% after 3–5 years follow-up in patients at higher risk (grade 2 and 3 VHWG). In one long-term series of complex abdominal wall reconstruction comparing absorbable scaffold with porcine cadaveric mesh, the absorbable mesh demonstrated better durability over five years, reinforcing the notion that a biosynthetic repair can remain intact beyond the resorption period (6).

The growing literature should not obscure the field's limitations. Most available studies are retrospective, industry-linked, single-arm, or based on heterogeneous populations that mix primary and incisional hernias, variable mesh planes, different component separation strategies, and broad contamination categories. Cost remains an additional challenge. LTABMs are generally less expensive than biologic matrices, which has supported their uptake as a cost-conscious alternative in complex reconstructions (7). However, they are usually still more expensive than standard permanent synthetics, so their value proposition depends on the clinical context, the probability of contamination-related morbidity, and the surgeon's estimate of the burden imposed by a permanent device. In health systems increasingly attentive to value-based care, these distinctions matter.

Patient selection is equally decisive. LTABMs are most compelling when the surgeon seeks reinforcement but wishes to avoid leaving permanent synthetic material in a field with elevated infection risk or uncertain tissue behavior (8). That does not mean it should replace permanent mesh in all moderate-risk patients, nor that it should be viewed as a surrogate for meticulous technique. Rather, it is best understood as a strategic material for selected reconstructions in which a temporary scaffold may align more closely with the biological reality of the operation.

LTABMs deserve a firm place in the contemporary discussion of abdominal wall surgery because they answer a clinically relevant question: can a repair be reinforced long enough to heal without committing the patient to a permanent prosthesis? The accumulated evidence suggests that, in selected patients, the answer may be yes. However, enthusiasm should remain disciplined. These materials are not a universal substitute for permanent synthetic mesh, nor have they eliminated the need for careful patient optimization, appropriate plane selection, and honest appraisal of recurrence risk. Their real contribution is more nuanced and perhaps more valuable: they expand the reconstructive options available to surgeons managing difficult abdominal walls in imperfect fields.
